# The Demands of Geometry on Color Vision

**DOI:** 10.3390/vision1010009

**Published:** 2017-01-12

**Authors:** Dale Purves, Chidambaram Yegappan

**Affiliations:** 1 Neuroscience and Behavioral Disorders Program, Duke-NUS Graduate Medical School Singapore, 8 College Road, Singapore 169857, Singapore; 2 Department of Neurobiology, Duke University Medical Center, Durham, NC 27708, USA; 3 Duke Institute for Brain Sciences, Duke University, Durham, NC 27708, USA

**Keywords:** perception, color circularity, opponency, trichromacy, spectral images, unique hues, four-color map problem

## Abstract

While studies of human color vision have made enormous strides, an overarching rationale for the circular sense of color relationships generated by two classes of color opponent neurons and three cone types is still lacking. Here we suggest that color circularity, color opponency and trichromacy may have arisen, at least in part, because of the geometrical requirements needed to unambiguously distinguish all possible spectrally different regions on a plane.

## 1. Introduction

Using sensory information to distinguish image regions that promote apt behavior in the physical world is generally assumed to be the broad purpose of animal vision. Whereas achromatic vision distinguishes regions on the basis of light intensity (luminance), color vision further distinguishes equiluminant regions on the basis of differences in the distribution of spectral energy, giving animals with this ability a behavioral advantage in dealing with objects and conditions in the world [1,2,3].

**Figure 1:**
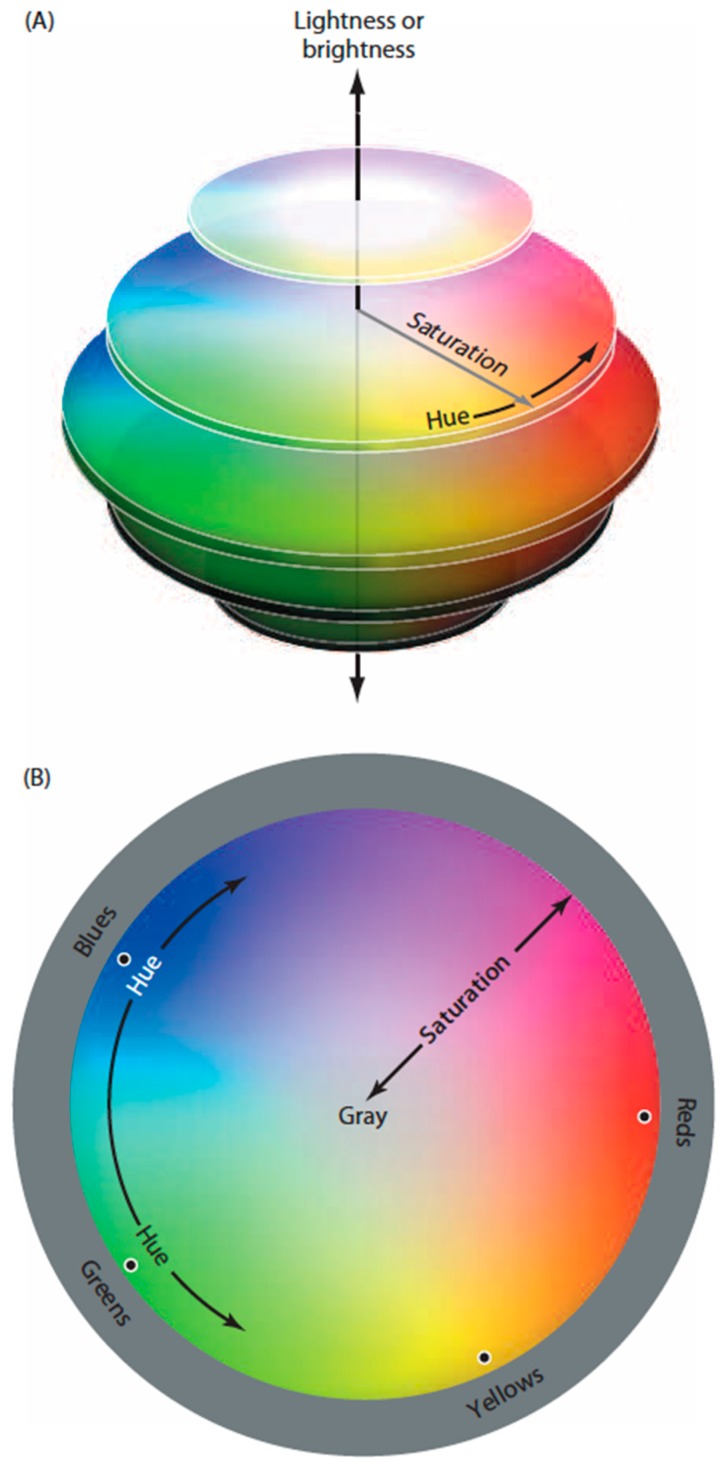
Diagram of human color perception. (**A**) A representation of human “color space”; (**B**) A single cross-sectional plane from the diagram in (**A**). When asked to arrange a large number of equiluminant surfaces that vary in hue such that the color differences among them are minimal, subjects arrange them in a closed loop that comprises four basic color categories (reds, greens, blues and yellows), each defined by a particular hue (black dots) that has no admixture of the neighboring color classes (e.g., a red surface seen as having no appreciable yellowness or blueness). Although there are many other named color groups (oranges, aquamarines, etc.), these are always seen as mixtures of the two of the four primaries. As indicated, the location of the unique hues (black dots) are not orthogonal in perceptual color space, nor is the closed loop determined psychophysically a literal circle as shown in the diagram.

Despite a wealth of anatomical, electrophysiological and psychophysical evidence about color vision, several general questions remain. A puzzle since Newton’s pioneering studies is why when asked to arrange objects with a full range of spectral qualities (e.g., Munsell chips) such that their apparent colors are minimally different, the result is a closed continuum (Figure 1) [4,5]. Equally perplexing is why we perceive a color gamut based on four color classes—reds, greens, blues and yellows—each defined by a unique hue that has no apparent admixture of the other three color classes [6,7,8,9]. And while it has long been known that color vision is mediated by the spectral sensitivities of short, medium, and long-wavelength cones whose output is processed by red-green and blue yellow opponent neurons [7,8,10,11,12], why these particular properties have evolved in humans is also incompletely understood [7,11,13].

Although the retina is a curved surface, the information it captures (the retinal image near the fovea) is generally thought of as a two-dimensional (2D) plane. Effectively distinguishing spectral differences on a plane presents at least two geometrical challenges. First, to achieve high resolution, different perceived colors must be ascribed to all spectrally different points on the retinal image plane. Second, perception must entail a number of color classes sufficient to ensure that adjoining regions on the plane will not be conflated if they are spectrally different. This latter challenge is called the “four-color map problem”, referring to the empirical minimum of four colors needed to ensure that countries on a map are always distinguishable (the “problem” in this phrase refers to the logic underlying this requirement, which took more than a century to verify) [14,15,16]. The relevance of this geometrical requirement to color vision is that at least four perceived color classes would be needed to resolve the same cartographic concern in the retinal images [17].

The purpose of the present article is to examine whether perceptual color circularity, color opponency and physiological trichromacy could be related consequences of a need to meet these geometrical demands.

## 2. Results

### 2.1. Geometrical Demands for Making Regional Distinctions in One Dimension

To appreciate the geometrical requirements that must be met in order to distinguish all possible regions of planar images by color percepts, consider the simpler challenge of distinguishing all possible regions in a hypothetical one-dimensional image.

**Figure 2:**
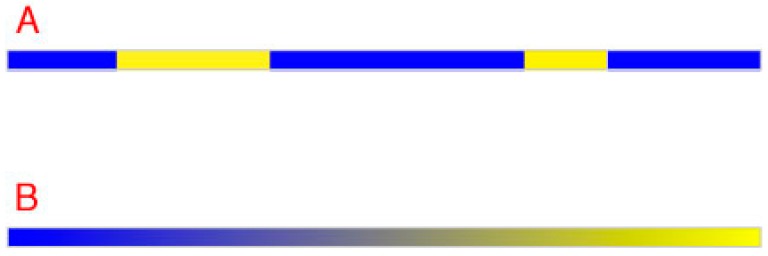
Distinguishing neighboring regions in one dimension. (**A**) Two different qualities (blue and yellow in this example) suffice to distinguish any number of neighboring regions from one another along a line; (**B**) However, a continuum is needed when points must be distinguished according to relative spectral differences at equiluminance. Notice that the balance point in a color continuum would be perceived is neither one color nor the other (i.e., neither blue nor yellow but a shade of gray).

From a geometrical perspective, two different qualities are all that is needed to distinguish any number of regions from their neighbors in a linear image (Figure 2A). Thus, in this hypothetical one-dimensional (1D) scenario, color vision could distinguish equiluminant regions by assigning two different color percepts to different spectra. Ultimately, however, a continuum would be needed, as shown in Figure 2B. Since the number of different spectra at any given level of luminance is limited only by the ability of the visual system to distinguish spectral variations, color vision would presumably evolve as many equiluminant perceptions as this constraint allowed (see Figure 1B). If points (i.e., the smallest regions resolved by human visual acuity) were arranged according to minimal differences among spectra, the result would require a continuum of two equiluminant color qualities, as indicated. The perceived difference between the extremes of such a 1D color space would be maximal, with a colorless intermediate sensation (gray) that was equally different from the sensations elicited by the two extremes. The continuum in the figure thus represents gradual spectral deviation in two opposite directions from a colorless balance point, with the maximum deviation eliciting the perceptions of a unique hue, e.g., a blue with no yellow or vice versa in this example. In sum, given the rules of geometry, two color classes defined by two unique hues are sufficient to distinguish all the points in a 1D image.

### 2.2. Quantification

Quantification of these spectral differences as vectors in 1D space is straightforward. Just as a line that extends from a null point in two directions can be specified by a pair of opposing direction vectors, such the *x* and −*x* vectors of a graphical axis, the spectral continuum in Figure 2B can be described by vectors (see Methods). The direction of each position vector from the null vector indicates the dominant color class at that location, and the distance from null vector indicates the relative contributions of the two unique hues. As the extremes of opposing direction vectors, the unique hues are necessarily opponent colors. The corresponding direction vectors indicate spectral deviation from the uniform distribution of energy at the color neutral null vector, and the distance between any two position vectors indicates the perceived spectral difference between them.

### 2.3. Biological Significance

**Figure 3:**
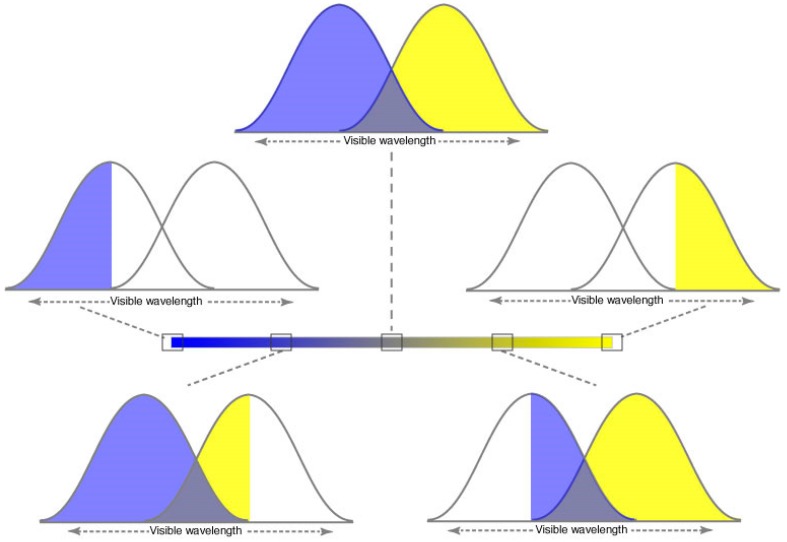
Color specification in 1D achieved by two opposing photoreceptor types. *Top panel*. Hypothetical cone sensitivity curves. When stimulated equally, the perceptual result would be color neutrality. *Middle panels*. When the response of one of the photoreceptor types is maximal and the other minimal, the perception would be that of unique blue (**left**) or unique yellow (**right**). *Bottom panel.* Stimulation that would give rise to predominantly blue (**left**) or yellow (**right**) perceptions along the color continuum in Figure 2B.

In terms of photoreceptors, two cone types with overlapping spectral sensitivities could generate these distinctions (Figure 3). The result would lead to the perceptual experience of human dichromats, and the majority of other mammals that posses only two types of cones (see Discussion) [18]. Thus individuals who lack L cones (protanopes) see spectral distributions that would have generated red or green for trichromats as shifts in brightness and/or saturation based on blue and yellow color classes, with a reduced color gamut. Similar findings have been reported for deuteranopes and tritanopes [19].

### 2.4. Geometrical Demands for Making Regional Distinctions in Two Dimensions

**Figure 4:**
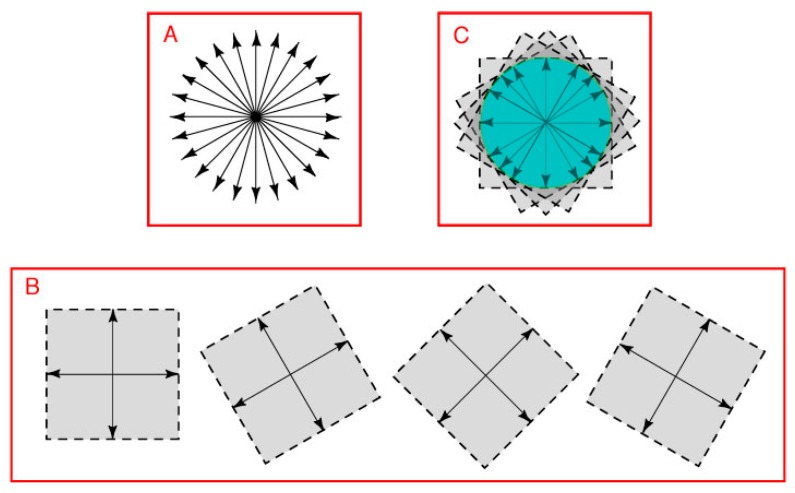
Distinguishing different spectral regions in a two-dimensional image. (**A**) A two-dimensional vector space extends in all directions from its null point; (**B**) Vector addition based on any two axes in the plane defines position vectors in all directions (four of the many arrangements possible are shown as examples); (**C**) The position vectors held in common by the full set of direction vectors would be necessarily be bounded by a closed loop, as indicated by the area in green. Notice that the loop would form a circle only if the vectors were all the same length, which the argument here does not require.

As in a hypothetical 1D image, equiluminant color perceptions arising from a plane can be represented by direction and position vectors. However, whereas 1D space extends in only two directions, 2D space extends in *all* possible directions from a null point (Figure 4A). Accordingly, position vectors in 2D must be expressed by two pairs of opposing direction vectors, as in the (−*x*, *x*) and (−*y*, *y*) axes that define any Cartesian co-ordinate system.

In vector addition (see Methods), two pairs of opposing direction vectors define the position vectors in all possible directions (Figure 4B). Of the many possible combinations, any two pairs of opposing direction vectors can be used to define the position vectors in all directions. The vector space defined by all possible sets of opposing direction vector pairs thus forms a closed loop that bounds the portion of the plane held in common (Figure 4C). Although not shown, the argument is the same whether or not the two pairs of opposing direction vectors are orthogonal.

### 2.5. Geometrical Rationale for Color Opponency

Given that the space held in common by all sets of opposing direction vector pairs in Figure 4C is bounded by a recursive perimeter, it follows that distinguishing points on a plane according to relative spectral differences would obey the same geometrical requirements.

**Figure 5:**
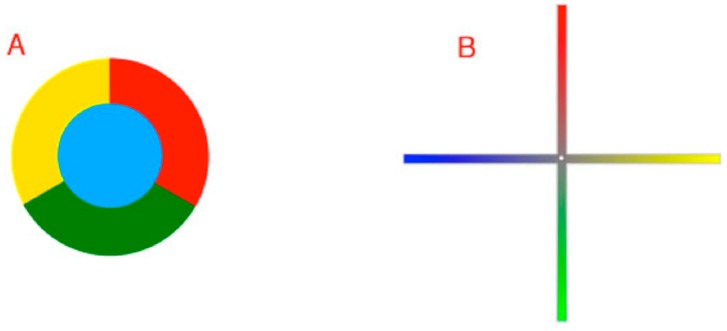
The four-color demand for regional distinctions on a map and the color opponency that would be needed. (**A**) The demand in 2D geometry refers to the fact that abutting regions on a plane cannot be distinguished using fewer than four colors. The diagram shows a simple example which makes clear that these regions could not be distinguished from one another using fewer than four colors. If this pattern were an equiluminant retinal image, the four regions would need to elicit color sensations that could distinguish any possible hue within these four color classes (reds, greens, blues and yellows); (**B**) Two opposing color quality pairs (direction vectors) extending from a null point (white dot) would be sufficient to address this issue, at the same time as differentiating all points (position vectors) on a plane by a gamut of color sensations. Note that the opponent axes would not have to be orthogonal.

In a two-dimensional (2D) space, however, the evolution of color vision would also have to contend with the geometrical demands apparent in map making (Figure 5A). That is, a minimum of four color classes is needed to distinguish all possible regions on a plane—i.e., all spectrally different equiluminant points in a 2D image. This goal could be met by two pairs of direction vectors that gave rise to four color classes defined by four unique hues that were pair-wise opponents (Figure 5B).

### 2.6. Geometrical Rationale for Color Perception as a Closed Loop

The demands of 2D geometry could also explain how the ability to order all equiluminant but spectrally different points on a plane according to minimal apparent differences leads to the closed continuum of hue sensations illustrated in Figure 1. Since the perimeter of a 2D space that extends equally in all directions is recursive (see Figure 4), the equiluminant sensations associated with the position vectors that lie between the opposing direction vectors in Figure 4B will also define a closed loop, with spectrally distinct points defined by vector addition based on the four representative spectral directions. Thus only four color classes are needed, although the number of perceived and named hues within each class can be large.

### 2.7. Geometrical Rationale for Retinal Trichromacy

This geometrical argument does not, however, indicate whether trichromacy could also be related to meeting the demands of plane geometry. On the contrary, from the constraints described so far one would expect that a least *four* cone types representing the two opponent axes would be needed to generate the four color classes illustrated in Figure 1 and Figure 5B. How, then, could the three cone types in humans and Old World monkeys generate the four color classes needed to distinguish all the equiluminant but spectrally different points on a plane?

**Figure 6:**
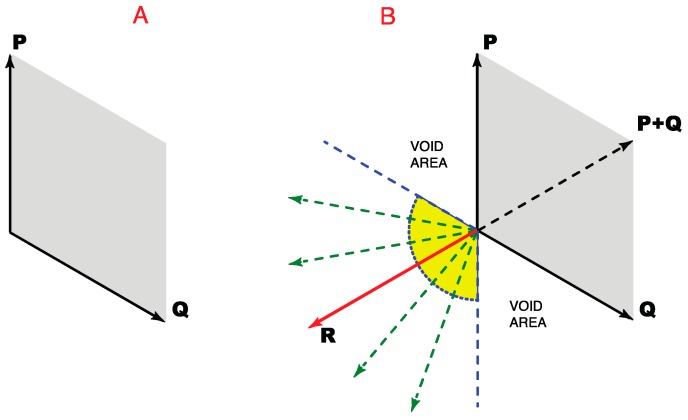
How three direction vectors can specify all possible locations (position vectors) on a plane. (**A**) Two non-opposing direction vectors (P and Q) could, by vector addition, specify all the position vectors that lie between them (the gray parallelogram); (**B**) A third direction vector, if appropriately positioned, would allow the resulting triad of vectors (P, Q and R) to specify locations in all directions. To deal with the four-color map demand, however, the third direction vector would have to oppose the combined influence of other two direction vectors, i.e., vector P + Q indicated by the dashed black line.

As shown in Figure 6A, the addition of any two non-opposing direction vectors can define all position vectors for all the directions that lie between them (see Methods). Accordingly, three direction vectors could, in principle, define all the position vectors on a plane. To do so, however, two of the three direction vectors would have to be non-orthogonal. As shown in Figure 6B, to satisfy these further geometrical demands, a third vector would have to lie within the area between the dashed blue lines.

Moreover, unless the third direction vector (vector R in Figure 6B) had a specific direction with respect to the two other vectors, only three color classes would arise, an arrangement that would be unable to contend with the four-color map concern illustrated in Figure 5A. Thus the third direction vector R would have to oppose the combined influence of the other two direction vectors (direction vector P + Q). An exception would be when vectors P and Q are equal. In this case, they would generate a null vector when vector R equals the combined influence of P and Q, as with the opposing direction vectors in Figure 5B. Thus P and Q are opponents only when direction vector R opposes the vector sum P + Q. In this way the second opponent pair needed to resolve the four-color map issue would be provided.

**Figure 7:**
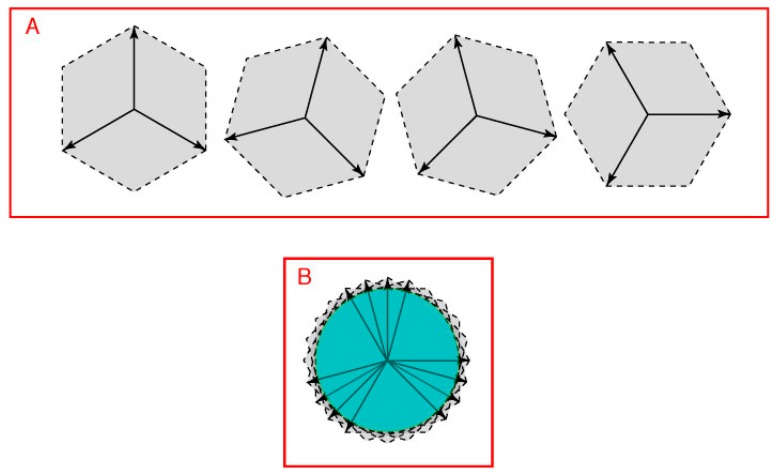
A closed 2D space defined by three direction vectors. (**A**) Vector addition based on three direction vectors placed appropriately (see Figure 6) would define position vectors in all directions on a plane (only four of many possible arrangements are shown as examples); (**B**) The position vectors held in common by the full set of such direction vectors form a circular space (green area) in this example.

Each of the four unique hues would correspond to the greatest deviation of spectral energy from the null vector of these directional extremes, i.e., the extremes of P, Q, R and P + Q in Figure 6. Intermediate color percepts would arise from the graded distribution of spectral energy between the extremes, as indicated by the gradients in Figure 5B. As shown in Figure 7, position vectors that lie between the three direction vectors in Figure 6B define a closed loop (cf. Figure 1 and Figure 4C). The interaction of the two pairs of opposing direction vectors in Figure 6 (P vs. Q, and R vs. P + Q) would be analogous to interaction of L vs. M cones, and S vs. L + M cones.

In short, the three cone types in humans and Old World monkeys may represent the minimum number of color receptors needed to generate the four color classes capable of distinguishing all resolvable equiluminant regions on a plane, at the same time resolving the need for four color classes to make unambiguous planar maps. The same argument would also explain why the four unique hues in human color space are not located at orthogonal extremes (see Figure 1B).

## 3. Discussion

The argument we have presented suggests that color circularity, color opponency, and trichromacy are related consequences of efficiently meeting the geometrical demands needed to distinguish all spectrally different points on the retinal image plane.

While this reasoning offers a unifying rationale for perceptual and physiological phenomena whose purposes are otherwise unclear, it also raises a number of questions. These issues and some speculative answers are as follows.

### 3.1. Why Are Red, Green, Blue and Yellow Perceptual Primaries?

The argument so far does not explain why the four primary color directions are red, green, yellow and blue, i.e., why visible spectra elicit these particular color classes.

Although a geometrical framework does not address this question, it nonetheless suggests an explanation. Just as the bias of magnetic north fixes the cardinal axes in geography, there are presumably biases in human spectral experience that would have determined the three direction vectors P, Q and R in Figure 6. The most obvious candidate for such biases is the distribution of light reflected from natural surfaces. Empirically, the reflectance efficiency functions of natural surfaces give rise to three spectral groups: foliage in the yellow-green region (~557–574 nm); “earths” in the yellow-orange region (~576–589 nm); and water, sky and distant objects in the blue region (~459–486 nm) [20]. These biases accord with the peak sensitivities of the three cone types, which in turn accord with the argument in Figure 6.

This agreement, however, does not explain why the combined influence of P and Q opposes R rather than the other options (i.e., Q and R opposing P, or P and R opposing Q). One possibility is a bias arising from the greater overlap of the spectral ranges of foliage and earth than from the overlap of either of these ranges with the blue range.

Finally, whereas that some primitive cultures lack names for these four color categories [21], the argument we outline implies that this deficiency in vocabulary is unlikely to arise from any differences in human visual physiology.

### 3.2. Why Can the Black-White Axis Not Serve as an Opponent Color Axis?

Since the perception of grays ranging from unique black to unique white forms an opponent axis, another question that arises is why this gray scale axis could not interact with a single color axis to distinguish all possible spectral points on a plane, at the same time resolving the four-color map problem.

The reason is that the color and gray scale axes concern different categories of information—light intensity versus the distribution of spectral energy—which are independent and lead to different sensory qualities (lightness versus hue). Although these domains can influence each other [7,22], like sensory qualities in other systems (e.g., pitch and loudness in audition) they are not combined in perception.

### 3.3. What about Other Rationales for these Phenomena?

Several plausible rationales for color opponency and trichromacy have been proposed. Opponency, which has been validated both psychophysically [12] and electrophysiologically [23,24] at several levels of the primary visual pathway in experimental animals, has been suggested to enhance the encoding of natural scene spectra [25], and/or to optimize the transfer of trichromatic color information [26,27]. By analogy with spatial sinusoids, trichromacy has also been interpreted in terms of comb-filtered spectra [28].

The difference in the present argument is that a geometrical explanation of color phenomenology is predicated on the advantages of distinguishing equiluminant image points. From this perspective, whatever else they may accomplish, opponency, color circularity and trichromacy are manifestations of resolving fundamental geometrical problems that would have to be addressed one way or another for the evolution of effective color vision.

### 3.4. What about Reports from Human Dichromats?

Another concern is the partial conflation of the trichromatic gamut in human dichromats, who retain some ability to perceive and identify colors that would be expected only in trichromats. For example, protanopes and deuteranopes show adaption to long wavelength light [29] and use the terms “red” and “green” as well as “blue“ and “yellow“ to describe what they see [30]. Montag and Boynton [31] have shown further that rods may contribute to these abilities. These further facts about color perception are not explained by the arguments here.

### 3.5. How Does the Argument Account of Color Metamers?

A final question concerns metameric stimuli—i.e., different distributions of spectral energy that elicit the same perceived colors in psychophysical matching tests. Such stimuli arise because the overlapping sensitivities the three human cone types allow similar relative activation by spectrally different stimuli. Since metameric image regions would elicit the same color percepts, metamers belie the idea that all spectrally different regions in arbitrarily complex images can be distinguished.

Although the question remains, the metameric stimuli used in colorimetry experiments are rare in natural images [32]. Thus the evolution of color vision may simply have ignored the legitimate conceptual problem posed by metamers.

## 4. Methods

**Figure 8:**
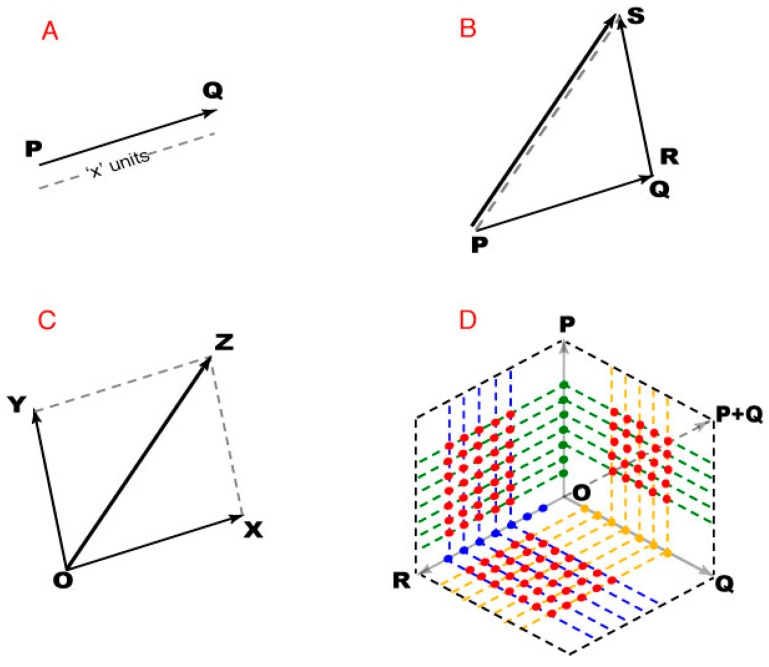
Using vector addition to describe spectral images. (**A**) PQ represents a vector directed from point P to point Q, the initial and terminal points of the vector. The magnitude of PQ thus indicates “x” units acting in that direction; (**B**) The tip-to-tail method vector addition, showing how PS is generated from vectors PQ and RS; *(***C**) Addition of vectors OX and OY using the parallelogram law of vector addition; (**D**) A 2D vector space with point O as origin. The green, yellow, and blue dots represent the end points (position vectors) of the direction vectors P, Q and, respectively. The red dots end points of position vectors determined as vector sums of the position vectors corresponding dotted lines.

As illustrated on Figure 8, we used vectors to describe the geometry of spectral images (to avoid any confusion, note that these diagrams are not a perceived color space). By the same token, the arguments here depend on the logic of plane geometry and not on any partiulcar metric of color space.

A plane is a 2D vector space in which any point can be specified as the position vector of that point with respect to an origin (Figure 8A). Thus the position vector of the origin is a null vector whose initial and terminal points are coincident, with zero magnitude and no direction. The arguments in the Results depend on vector addition. If PQ and RS are any two non-zero vectors, their addition leads to a third vector PS that is different from both PQ and RS (Figure 8B). The magnitude and direction of PS depends on the magnitude and direction of vectors PQ and RS. The addition of vectors with PQ and/or RS as null vectors is still valid, but the resultant vector PS may be equal to both or one of the addends.

When adding vectors, the initial point of the second vector is placed at the terminal point of the first, resulting in a vector co-initial with the first vector and co-terminus with the second vector. Since vector addition is commutative, either of the addends can be treated as the first vector. For addition involving more than two vectors, stepwise addition was performed by taking two vectors at a time, with the resultant vector of a pair being one of the addends for the subsequent addition.

Finally, when two vectors are adjacent sides of a parallelogram, their vector sum is given by the diagonal of the parallelogram (Figure 8C). This parallelogram rule gives the same result as the tip-to-tail method, except that it operates on co-initial addends, and was used to define position vectors based on other position vectors (Figure 8D). When two direction vectors are represented by adjacent sides of a parallelogram, the included position vectors defined all the position vectors within that parallelogram (i.e., the black dotted lines connecting all the direction vectors in Figure 8D).

## 5. Conclusions

To be optimally successful, color vision must assign distinct percepts to all possible arrangements of spectrally different regions on a plane, while at the same time contending with the need of four color classes to make any planar map unambiguous. Here we suggest that the circularity of color perception, color opponency, and trichromacy have evolved at least in part as related ways of meeting these geometrical demands. This perspective provides a unified rationale for perceptual and physiological phenomena that are otherwise difficult to explain.

## References

[1] Land M.F., Nilsson D.-E. (2002). Animal Eyes.

[2] Mollon J.D., Cronly-Dillon J.R., Gregory R.L. (1991). Uses and evolutionary origins of primate color vision. Evolution of the Eye and Visual System, Vision and Visual Dysfunction.

[3] Frome F.S., Buck S.L., Boynton R.M. (1981). Visibility of borders: Separate and combined effects of color differences, luminance contrast, and luminance level. J. Opt. Soc. Am..

[4] Newton I. (1952). Opticks: Or, a Treatise of the Reflexions, Refractions, Inflexions and Colors of Light.

[5] Munsell A.H. (1905). A Color Notation.

[6] Evans R. (1948). An Introduction to Color.

[7] Hurvich L. (1981). Color Vision.

[8] Wandell B. (1995). Foundations of Vision.

[9] Kuehni R.G., Schwarz A. (2008). Color Ordered: A Survey of Color Systems from Antiquity to the Present.

[10] Macaluso C., Lamedica A., Baratta G., Cordella M. (1996). Color discrimination along the cardinal chromatic axes with VECPs as an index of function of the parvocellular pathway. Correspondence of intersubject and axis variations to psychophysics. Electroencephalogr. Clin. Neurophysiol..

[11] Kaiser P., Boynton R. (1996). Human Color Vision.

[12] Hurvich L.M., Jameson D. (1957). An opponent-process theory of color vision. Psychol. Rev..

[13] Hubel D.H. (1988). Eye, Brain, and Vision.

[14] Cayley A. (1878). On the colouring of maps. Proc. Lond. Math. Soc..

[15] Ore O. (1967). The Four-Color Problem.

[16] Appel K., Haken W. (1977). The solution of the four-color- map problem. Sci. Am..

[17] Purves D., Lotto B., Polger T. (2000). Color vision and the four-color-map problem. J. Cogn. Neurosci..

[18] Neumeyer C., Cronly-Dillon J.R., Gregory R.L. (1991). Evolution of color vision. Uses and Evolutionary Origins of Primate Color Vision.

[19] Brettel H., Viénot F., Mollon J.D. (1997). Computerized simulation of color appearance for dichromats. JOSA A.

[20] Hendley C.D., Hecht S. (1949). The colors of natural objects and terrains, and their relation to visual color deficiency. JOSA.

[21] Berlin B., Kay P. (1969). Basic Color Terms: Their Universality and Evolution.

[22] Helmholtz H.L.F.V. (1924). Helmholtz’s Treatise on Physiological Optics. Opt. Soc. Am..

[23] Chatterjee S., Callaway E.M. (2003). Parallel colour-opponent pathways to primary visual cortex. Nature.

[24] Shapley R., Hawken M. (2002). Neural mechanisms for color perception in the primary visual cortex. Curr. Opin. Neurobiol..

[25] Schluppeck D., Engel S.A. (2002). Color opponent neurons in V1: A review and model reconciling results from imaging and single-unit recording. J. Vis..

[26] Lee T.W., Wachtler T., Sejnowski T.J. (2002). Color opponency is an efficient representation of spectral properties in natural scenes. Vis. Res..

[27] Buchsbaum G., Gottschalk A. (1983). Trichromacy, opponent colours coding and optimum colour information transmission in the retina. Proc. R. Soc. Lond. Ser. B Biol. Sci..

[28] Barlow H.B. (1982). What causes trichromacy? A theoretical analysis using comb-filtered spectra. Vis. Res..

[29] Scheibner H.M.O., Boynton R.M. (1968). Resiidual red-green discrimination in dichromats. J. Opt. Soc. Am..

[30] Wachtler T., Dohrmnn U., Ertel R. (2004). Modeling color percepts of dichromats. Vis. Res.

[31] Montag E.D., Boynton R.M. (1987). Rod influence in dichromatic surface color perception. Vis. Res..

[32] Foster D.H., Amano K., Nascimento S.M.C., Foster M.J. (2006). Frequency of metamerism in natural scenes. J. Opt. Soc. Am. A.

